# Brillouin expanded time-domain analysis based on dual optical frequency combs

**DOI:** 10.1038/s41377-024-01499-x

**Published:** 2024-07-02

**Authors:** Jae Hyeong Youn, Kwang Yong Song, Sonia Martin-Lopez, Miguel Gonzalez-Herraez, María R. Fernández-Ruiz

**Affiliations:** 1 Dept. of Physics, Chung-Ang University, Seoul, Korea; 2 Dept. of Electronics, Universidad de Alcalá, Madrid, Spain

**Keywords:** Fibre optics and optical communications, Optical sensors

## Abstract

Brillouin Optical Time-Domain Analysis (BOTDA) is a widely-used distributed optical fiber sensing technology employing pulse-modulated pump waves for local information retrieval of the Brillouin gain or loss spectra. The spatial resolution of BOTDA systems is intrinsically linked to pulse duration, so high-resolution measurements demand high electronic bandwidths inversely proportional to the resolution. This paper introduces Brillouin Expanded Time-Domain Analysis (BETDA) as a modified BOTDA system, simultaneously achieving high spatial resolution and low detection bandwidth. Utilizing two optical frequency combs (OFCs) with different frequency intervals as pump and probe, local Brillouin gain spectra are recorded by their spectral beating traces in an expanded time domain. A 2-cm-long hotspot located in a 230 m single-mode fiber is successfully measured in the time domain with a detection bandwidth of less than 100 kHz using dual OFCs with tailored spectral phase, line spacing, and bandwidth.

## Abstract (web summary)

BETDA, using dual optical frequency combs, reduces pump-probe beating to low-frequency bands in an expanded time domain, unlike ordinary BOTDA which operates at high RF frequency bands.

## Graphical abstract

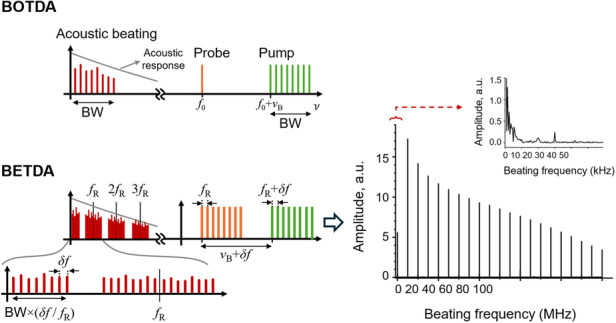

## Introduction

A distributed optical fiber sensor (DOFS) is capable of measuring the spatial distribution of one or more physical parameters (or measurands) at each and every point along a sensing fiber^1^. Today, DOFS systems have gained widespread usage, primarily for real-time monitoring of the structural integrity of expansive civil infrastructures and the changes in their environmental conditions. DOFS operate utilizing elastic or inelastic light scatterings within optical fibers, responding to various physical quantities such as strain^2^, temperature^3^, pressure^4^, vibration^5^, and acoustic impedance^6^. They serve as fundamental components in smart sensing systems, playing a pivotal role in the development of smart cities and factories.

Distributed Brillouin sensors, relying on acoustic phonon-induced light scattering, demonstrate distinct sensitivity to variations in both strain and temperature^7^. Over the years, Brillouin sensors have been developed across various platforms, including time-domain^8,9^, correlation-domain^10,11^, and frequency-domain^12,13^ systems utilizing spontaneous and stimulated scattering processes.

Brillouin optical time-domain analysis (BOTDA), a representative distributed Brillouin sensor operating in the time-domain, functions through the Brillouin amplification of a continuous probe by a pulse-modulated pump via stimulated Brillouin scattering (SBS). The frequency offset between the pump and probe maximizing the Brillouin amplification, known as Brillouin frequency shift (*ν*_B_), is measured at each position. This frequency shift linearly depends on the strain and temperature variations. Over the past three decades, research efforts aimed at enhancing the performance of BOTDA systems have yielded considerable success, and these advancements encompass key sensing parameters such as sensing range^14–16^, spatial resolution^17–20^, sensing speed^21,22^, and accuracy^23,24^. Although BOTDA systems necessitate two-end access to a sensing fiber due to counter-propagation of pump and probe, they offer advantages of long sensing distances spanning hundreds of kilometers^16^ and high spatial resolutions at the order of centimeters^18–20^. These advantages primarily stem from the larger signal compared to Brillouin reflectometry utilizing spontaneous Brillouin scattering^9^.

The spatial resolution of BOTDA systems is fundamentally linked to the duration of the pump pulse. In earlier works, these resolutions were typically limited to approximately 1 meter, primarily due to the spectral broadening of the pump pulse^25^. This broadening deteriorates both the accuracy of Brillouin frequency determination and the signal amplitude, especially when it considerably exceeds the Brillouin gain bandwidth (~30 MHz). To mitigate this effect while maintaining high spatial resolutions, several innovative schemes have emerged, such as pulse pre-pump BOTDA (PPP-BOTDA)^17^, differential pulse-width pair BOTDA (DPP-BOTDA)^18^, Brillouin echo^19^, and Brillouin dynamic grating (BDG) BOTDA^20^. These approaches enable centimeter-level spatial resolutions. However, achieving such resolutions commonly demands higher detection bandwidths to accommodate their pulse-based nature. For instance, achieving a spatial resolution of 10 cm requires a pulse duration time of 1 ns^17,20^ or pulse rising/falling edges of sub ns^18,19^, which necessitate a detection bandwidth exceeding 1 GHz and a data acquisition (DAQ) sampling rate exceeding 2 GSa/s. Consequently, this raises the system cost and noise levels.

Recently, time-expansion reflectometry has emerged as a solution to address the inverse relationship between spatial resolution and detection bandwidth in time-domain systems^26,27^. This approach can be seen as a successful implementation of dual-comb spectroscopy (DCS) within a distributed sensing system. DCS is a powerful spectroscopic technique that leverages the frequency resolution, accuracy, broad bandwidth, and brightness of optical frequency combs for ultrahigh-resolution and high-sensitivity broadband spectroscopy^28^. By employing multi-heterodyne interference between two comb lines, DCS facilitates the down-conversion of optical frequencies onto the radio-frequency (RF) domain, effectively combining the advantages of conventional broadband spectroscopy and tunable laser spectroscopy into a single platform^28,29^. This innovative technique enables a high spatial-resolution time-domain Rayleigh system to operate with a detection bandwidth tens of thousands of times smaller than the one typically required for achieving the same resolution^27^. The time-expansion method utilizes two optical frequency combs (OFCs) with identical spectral phases and slightly different line spacings, acting as probe signal and local oscillator. By interfering with the local oscillator, the spectral components of the back-scattered probe are down-converted into a narrow bandwidth within the radiofrequency (RF) band. The specific phase coding used in the two combs implies that the impulse response of the fiber is directly recovered in the time domain, however over an extremely expanded time axis. This approach, however, has only been used in the determination of a linear, elastic spontaneous scattering (Rayleigh). Extending the concept to the determination of a nonlinear stimulated scattering poses a significant challenge.

In this study, we introduce a novel approach termed Brillouin Expanded Time-Domain Analysis (BETDA) that extends the time expansion concept to BOTDA. This innovative technique enables the simultaneous achievement of significantly reduced detection bandwidth and high spatial resolution. For the Brillouin interaction, we utilize two counterpropagating pump and probe OFCs exhibiting central frequencies around the fiber Brillouin frequency shift, and with a minute difference in comb line spacing. The information on the local Brillouin gain by SBS between the pump and probe is spectrally down-converted by the beating between the two combs.

Time expansion techniques in Rayleigh sensors^26,27^ utilize a dual comb setup where the interrogation is linear. In this setup, the probe and local oscillator combs beat at the detector level. In contrast to Rayleigh sensors, in Brillouin analysis systems like BETDA, the beating between pump and probe combs occurs within the fiber itself through the mediation of acoustic phonons. This introduces unique considerations, including the temporal response of the acoustic phonons, which must be accounted for in the design of the combs. The resulting Brillouin gain spectrum is strongly influenced by the relative phase of each comb line. Developing a comprehensive theory for BETDA is therefore needed to properly design the interrogation combs and interpret the acquired Brillouin gain spectra, as we will show below.

Our work represents the first demonstration of a time-expanded distributed sensing system utilizing an inelastic light scattering caused by nonlinear optical phenomenon, and also introduces a novel approach to implementing a high-spatial resolution BOTDA system without experiencing broadening of the BGS.

## Results

**Fig. 1:**
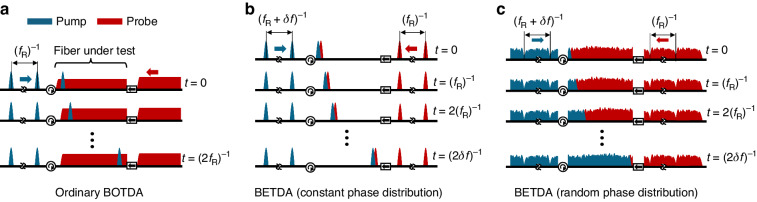
Arrangement of the propagation of pump and probe waves in different Brillouin optical time-domain analysis-based configurations. **a** Ordinary BOTDA; **b** BETDA with comb lines of constant phase, and **c** BETDA with comb lines of random phases

Figure 1a–c schematically show the propagation of pump and probe waves in ordinary BOTDA, BETDA with comb lines of constant phase, and BETDA with comb lines of random phases, respectively. It is worth mentioning that the case of BETDA with comb lines of constant phase corresponds to the pulse modulation of both pump and probe waves with slightly different repetition rates to sweep the position of their overlap over time. In the case of BETDA with comb lines of random phases, the overlapped pattern resulting from the random intensity variations of the pump and probe is swept along the position over time.

**Fig. 2:**
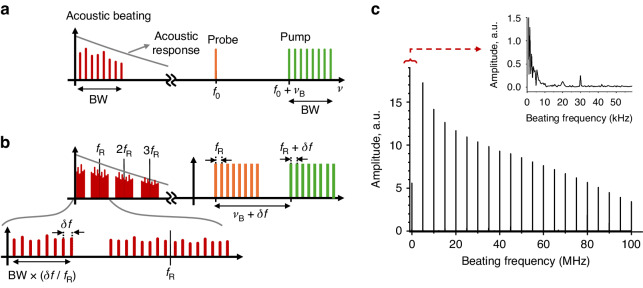
Spectral-domain representations of wave interaction. **a** Conventional BOTDA based on a pulsed pump. **b** Proposed BETDA using dual OFCs. **c** Spectral beatings between dual OFCs. The inset in panel **c** provides a zoomed view of the 1st Nyquist zone

Figure 2 illustrates the spectral-domain representations of the wave interaction for both ordinary BOTDA based on a pulsed pump and the proposed BETDA based on dual OFCs. The temporal beating of pump and probe that yields the acoustic wave is equivalent to the convolution of the spectra in the frequency domain. In the conventional BOTDA, the spectral width of the acoustic wave made by the interaction of a monochromatic probe and a pulsed pump will be the same as that of the pump as shown in Fig. 2a. In BETDA, the pump and probe are dual combs with a slight difference (*δf*) in comb line spacing as shown in Fig. 2b. In this case, groups of spectral lines will be clustered around the frequencies that are multiples of the probe line spacing, *f*_R_, the spacing within the clusters (usually termed Nyquist zones) being given by *δf*. The resulting beating spectrum will be low pass filtered by the characteristic BGS. In other words, higher order Nyquist zones will be increasingly attenuated by the acoustic response of silica. Since the receiver used in this technique has a detection bandwidth lower than *f*_R_*/*2, only the lower frequency terms (i.e., the 1st Nyquist zone) of the acoustic beating spectrum will be retained. Also, since *f*_R_ is much smaller than the natural Brillouin gain bandwidth, we can use a constant signal approximation for the gain suffered by the probe along each pulse roundtrip time (1*/f*_R_).

Note that the difference in order of magnitude between *f*_R_ and *δf* in the BETDA is selectable and can be easily in the order of thousands or tens of thousands. In other words, the detection bandwidth can be reduced to the hundreds of kHz range even for cm-scale resolutions implying GHz bandwidths in the pump and probe modulations. An example of the beating measured in a dual-comb configuration is shown in Fig. 2c, which displays an RF spectrum measured at the unfiltered output of the photodetector (BPD in Fig. 7). The parameters used in that figure are *f*_R_ = 5 MHz, *δf* = 500 Hz and the number of comb lines is N = 100. It is evident that the signals of the higher-order Nyquist zones are attenuated due to the low pass filtering imposed by the characteristic BGS. The inset is a zoomed view of the first Nyquist zone, and such signals exist equally in all Nyquist zones.

First, we conducted test measurements on a short length (approximately 10 m) of SMF, to verify the effects of dual OFCs on local BGS. Comparative measurements were carried out between OFCs with constant phase distribution and random phase distribution, both operating under the same peak power of the pump. Given that the dual OFCs employed in the BETDA are designed to down-convert the probe signal to the low-frequency domain, our attention is specifically directed to the beating between the comb lines of the same order. In this context, even if a random phase distribution is applied to the OFC, the relative phase between two comb lines of the same order consistently remains zero, as expected according to Eqs. (13) and (14) in the Materials and methods section.

**Fig. 3:**
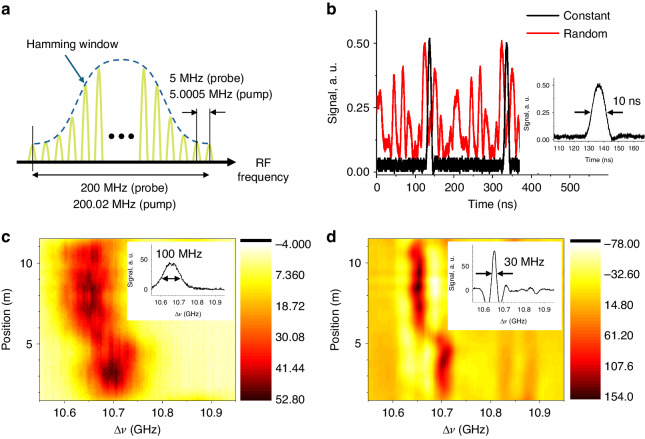
Experimental test of BETDA targeting a short range of 20 m. **a** Spectral configuration of RF combs. **b** Output waveforms of OFCs with constant (black) and random (red) phase distributions. 3D map of BGS along a 10 m SMF measured using dual OFCs with **c** a constant phase distribution and **d** a random phase distribution, respectively. The insets are examples of local BGS obtained at the position of 5 m

Figure 3a shows the RF spectrum configuration of dual OFCs used to test the effect of the phase distribution of the comb line. The full bandwidth of the OFC is 200 MHz (200.02 MHz) for probe (pump), consisting of 40 lines with the line spacing of 5 MHz (5.0005 MHz) for probe (pump), respectively. The offset in the comb line spacing (*δf*) is 500 Hz, and the bandwidth of the down-converted beating signal in the first Nyquist zone is 20 kHz. To suppress sidelobes of OFCs the intensity distribution of the induced RF spectrum is apodized by a Hamming window, resulting in effective reduction of bandwidth.

Figure 3b compares the output waveforms, i.e. temporal power variations, of the OFCs with different phase distributions for each comb line. In the scenario of a constant spectral phase distribution, the comb in the time domain is represented as a pulse train (depicted in black). The pulse duration is approximately 10 ns, and the period is 200 ns, resulting in a spatial resolution and measurement range of 1 m and 20 m, respectively. The effective duty cycle, considering the area of the envelope in the time domain, is calculated to be 3.9%. In the case of an OFC with a random phase distribution, random-like power variations (depicted in red) emerge. The effective duty cycle increases to 33.1%, approximately 8.5 times larger than that of the pulse. Since the peak power of BOTDA systems is fundamentally constrained by the onset of modulation instability^30^, it is anticipated that the random phase distribution secures a larger pump energy injected in the fiber over a measurement cycle, i.e. larger signal than in the case of constant phase distribution.

Figure 3c shows the 3D map of BGS along a 10 m single mode fiber (SMF) measured using dual OFCs with a constant phase distribution, and the inset is an example of local BGS obtained at the position of 5 m. Spectral broadening of approximately 100 MHz is observed as expected from the use of a pump pulse with a bandwidth broader than the intrinsic Brillouin gain bandwidth (30 MHz)^25^. Figure 3d is the 3D BGS map of the same optical fiber obtained using dual OFCs with a random phase distribution. As depicted in the inset, a narrow local BGS close to 30 MHz, the intrinsic Brillouin gain bandwidth, is observed. This outcome serves as clear experimental evidence of the restoration of the intrinsic BGS by randomizing the phase of comb lines in the two combs and reducing the detection bandwidth to within 20 kHz, as anticipated by Eq. (14) in the Materials and methods section.

During the measurement of probe traces, we observed low-frequency fluctuations attributed to slow intensity noise corresponding to the DC component in Eq. (14). These fluctuations could be mitigated through averaging and normalization processes during the acquisition of the BGS.

**Fig. 4:**
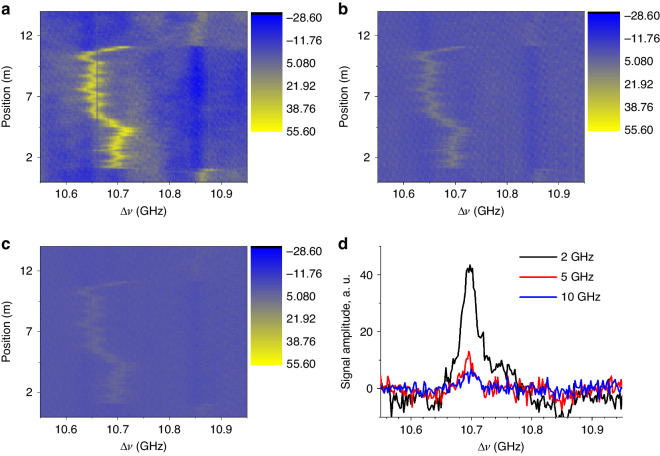
Brillouin maps and Brillouin gain spectra measured by dual combs with different bandwidths. Brillouin map measured by dual comb of **a** 2 GHz, **b** 5 GHz, and **c** 10 GHz, respectively. **d** Brillouin gain spectrum obtained by 2, 5, and 10 GHz bandwidth dual OFCs

Figure 4a–c present 3D maps of the BGS along the 10 m SMF, obtained by setting the number of comb lines to 400, 1000, and 2000, respectively. The comb line interval (*f*_R_) and its offset (*δf*) are fixed at 5 MHz and 500 Hz. The total comb bandwidths are 2 GHz, 5 GHz, and 10 GHz, corresponding to spatial resolutions of 5 cm, 2 cm, and 1 cm, respectively. While the distributions of BGS are observed approximately the same, the signal-to-noise ratio (SNR) decreases as the spatial resolution becomes higher. Figure 4d illustrates the BGS measured at the same position (near 3 m) with different spatial resolutions, where the relative signal magnitudes are 1.0, 0.29, and 0.15, respectively. Particularly noteworthy is the very low amplitude of the signal without a sharp peak for the 10 GHz comb bandwidth. This poor signal quality is attributed to the spectral proximity between the edges of the Stokes and Anti-Stokes bands, making it challenging to clearly separate the two bands with the DWDM filter. Based on these initial results, the target spatial resolution was set to 2 cm to ensure the normal operation of the OPS scheme explained in the Materials and methods section. We believe that polarization scrambling instead of the OPS could potentially enhance spatial resolution further. However, in this scenario, the measurement time would be doubled to maintain the same level of sensing accuracy.

**Fig. 5:**
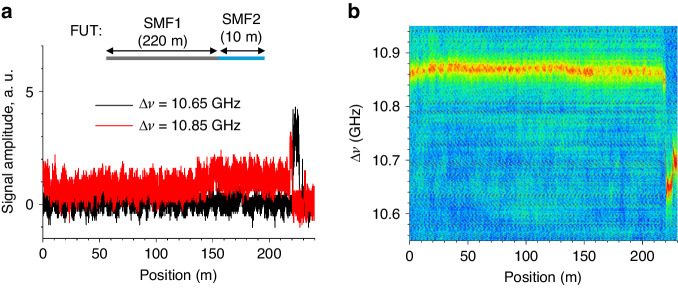
Experimental test of BETDA targeting a range of 230 m using dual comb with random phase distribution. **a** Probe traces obtained with different Δ*ν*’s, each equal to the *ν*_B_’s of two SMFs concatenated to compose a 230 m fiber under test (FUT). The inset illustrates the structure of the FUT. **b** 3D map of BGS constructed with 201 traces obtained by scanning Δ*ν* from 10.55 to 10.95 GHz

The OFCs used in our final measurements consist of 12,000 lines with random phase distribution. Each comb line is separated by *f*_R_ of 417 kHz, resulting in a total bandwidth of 5 GHz. According to the comb specification, the system achieves a spatial resolution of 2 cm and a measurement range of 248 m. The pump-probe interval offset, *δf*, is set to 8 Hz. In Fig. 5a, examples of probe traces obtained at different values of Δ*ν* (10.85 GHz and 10.65 GHz) are presented, corresponding to the approximate values of *ν*_B_ of the two SMFs concatenated to form a 230 m FUT. The acquisition time is expanded to 0.125 s, slightly larger than 52,125 (equal to *f*_R_ */ δf*) times the typical time (approximately 2.3 μs) required for measuring FUTs of the same length in a conventional BOTDA. The inset illustrates the configuration of the FUT consisting of approximately 220 m of conventional SMF (SMF1) and 10 m of special SMF (SMF2) enclosed with two metal wires wrapped in a plastic jacket. By applying current to a section of the wire of the cable, heating can be induced along a desired length of a few cm.

Figure 5b depicts the 3D map of BGS, composed 201 traces. Each trace was obtained after averaging 16 time-expanded traces for 2 s. Δ*ν* is scanned from 10.55 GHz to 10.95 GHz at 2 MHz steps. A uniform distribution with the *ν*_*B*_ of 10.85 GHz is observed in SMF1 from the fiber input to the position of 220 m. Beyond that, a non-uniform *ν*_B_ distribution appears due to strain induced by bending of the hard plastic jacket. The boundary between the two different fibers is clearly identified with a sharp drop at the position of 220 m.

**Fig. 6:**
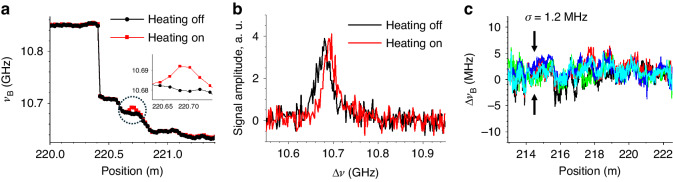
Perturbation detection. **a** Distributions of *ν*_B_ with and without heating current in a 2 cm test section of the FUT, focusing on the hotspot position. The inset show the zoomed view on the hotspot (dotted circle). **b** BGS comparison at the center of the hotspot with and without heating current. **c** Deviation of *ν*_*B*_ in the plots of repeated measurements. The standard deviation *σ* is calculated to be 1.2 MHz

In Fig. 6a, the map of *ν*_B_ along the fiber is presented, derived by fitting the local measured BGS with a Gaussian function. The position sampling step is approximately 2.0 cm (equal to 248 m/12500 points). Notably, when a current was applied to a 2 cm-long test section, inducing a temperature rise of about 10 degrees, a local shift in *ν*_B_ becomes evident at 220.7 m, as clearly depicted in the zoomed view of the inset. This change is further illustrated in Fig. 6b, displaying the BGS measured in the middle of the hotspot. The BGS distinctly indicates a spectral shift of approximately 10 MHz when heated, aligning with the anticipated thermal response of *ν*_B_ in a conventional SMF (typically around 1 MHz/^o^C at this wavelength). Figure 6c provides insight into the deviation of the repeatedly measured *ν*_B_, validating the accuracy of the distributed measurement. The calculated standard deviation averages at approximately 1.2 MHz.

## Discussions

We have introduced and successfully implemented a novel technique called Brillouin Expanded Time Domain Analysis (BETDA). The technique is based on the SBS interaction of two carefully engineered optical frequency combs with the same random spectral phase. Such scheme allows a direct recovery of the Brillouin gain time-domain trace of the fiber; however this can be done with a significantly reduced detection bandwidth compared to traditional BOTDA systems. The use of dual OFCs allowed us to achieve an impressive 2 cm spatial resolution with a detection bandwidth of less than 100 kHz, which implies a bandwidth reduction of 52,000 times compared to typical time-domain systems.

This implementation not only achieves bandwidth reduction, but also provides a narrowband BGS for high resolution sensing. This feature is again made possible thanks to the random phase distribution of comb lines. The use of a reduced electrical bandwidth together with an increased optical power (again deriving from the random spectral phase distribution of the pump and probe combs) entails an increase in the system SNR which stands in contrast to the conventional BOTDA pulsed modulation.

The achievement of centimeter-order spatial resolution presents a significant challenge in conventional BOTDA systems. A spatial resolution of 1 cm corresponds to a pulse duration of 100 ps (or rising / falling edge of less than 100 ps) and a spectral width of 10 GHz for the pump pulse in a BOTDA system. However, due to factors such as the onset time (a few nanoseconds) of acoustic phonons and the narrowband (~30 MHz) intrinsic BGS in optical fibers, achieving cm-order spatial resolution typically results in a significant reduction of Brillouin gain and substantial broadening of the BGS. To address these challenges, various enhancement techniques have been developed, including dark pulse, differential pulse-width pair (DPP), and Brillouin dynamic grating (BDG), to achieve spatial resolutions equal to or less than 2 cm^20,31,32^.

It is insightful to compare the performance of our work with that of former high-resolution BOTDA systems. In a dark-pulse-based BOTDA system (as reported in Ref. ^31^), a spatial resolution of 2 cm was demonstrated using an 80 m optical fiber, with an acquisition time of 7 min and a sensing error of about 1 MHz. In the DPP-BOTDA system (reported in Ref. ^32^), a spatial resolution of 2 cm was achieved with a 2 km optical fiber. The estimated acquisition time and sensing error were a few seconds and about 2 MHz, respectively. In the Brillouin dynamic grating approach (as reported in Ref. ^20^), a spatial resolution of 1 cm was demonstrated using a 20 m polarization-maintaining fiber, with an estimated acquisition time of less than 1 s and a sensing error of about 1 MHz. It is evident that the overall performance of our work – achieving a spatial resolution of 2 cm with a 230 m optical fiber, a sensing time of 400 s, and a sensing error of 1.2 MHz – is comparable to the dark-pulse approach, albeit slightly lower than that of the DPP-BOTDA and BDG-based systems, particularly in terms of sensing time. However, it should be emphasized that all former BOTDA systems with cm-order spatial resolution require at least 5 GHz detection bandwidth, which is 5×10^4^ times larger than our approach. Thus, our system presented here shows competitive performance with all these previous realizations while having extremely more modest detection and acquisition requirements.

Our dual-comb-based Brillouin sensor can be understood as a hybrid system combining elements of BOTDA and BOCDA. This interpretation arises from considering the time expansion in our system as analogous to the slow movement of the correlation peak (CP), which characterizes the sensing position in BOCDA. The key distinction between BOTDA and BOCDA lies in the analysis domain—specifically, “time-domain” versus “correlation-domain” analysis of the local BGS. In time-domain systems like BOTDA, BOTDR, and BETDA (as presented in this work), there exists a one-to-one correspondence between the received time trace and the sensing position. Conversely, in correlation-domain systems like BOCDA and BOCDR, the measurement position is determined by the position of CP, which is dictated by modulation parameters. Generally, there is no one-to-one correspondence between the received time trace and the sensing position in correlation-domain systems. However, there is some ambiguity when considering systems that exhibit both the CP and the one-to-one correspondence between time and position, as is the case with our technique. In such instances, these systems could be referred to as either modified BOTDA or modified BOCDA systems. In our work, the primary objective is to expand time and reduce detection bandwidth. Considering this objective, we believe it is reasonable to designate our technique as Brillouin “expanded time-domain” analysis (BETDA), focusing on the one-to-one correspondence between time and sensing position.

One can find common ground between our work and the BOCDA with sweeping CP (called DM-BOCDA), which introduces differential frequency modulation to the pump and probe waves using sine waves of different frequencies^33^. In DM-BOCDA, the CP continuously sweeps the sensing fiber, and the BGS is constructed by recording the time traces of the probe wave, similar to our approach. However, the DM-BOCDA scheme inherits limitations from traditional BOCDA systems. The sinusoidal direct current modulation introduces drawbacks such as strong intensity chirp in the optical spectrum and specific phase relations between spectral lines, resulting in similar noise substructures observed in BGSs of conventional BOCDA systems. Additionally, the achieved spatial resolution (about 80 cm) falls short of meeting the demands of high-resolution sensing. This limitation primarily stems from challenges associated with generating pump and probe waves in the time domain through direct modulation of a laser diode. In contrast, our proposed BETDA utilizes random-phase dual OFCs, enabling high spatial resolution and a narrow BGS while suppressing noise structures through random phase modulation of comb lines.

The primary advantage of our work lies in achieving high resolution and a narrow BGS while maintaining relatively high-speed readout and low detection bandwidth simultaneously. This combination is highly appealing for high-resolution systems. In comparison, ordinary BOTDA offers faster readout but exhibits a broad BGS and requires a higher detection bandwidth. BOFDA typically necessitates a higher detection bandwidth proportional to the spatial resolution, and BOCDA usually requires higher detection bandwidth and slower readout due to lock-in detection. Despite the advantages of our scheme, the generation stage becomes more expensive, and the readout speed is reduced compared to BOTDA.

Although the cost of a commercial multi-GHz-bandwidth arbitrary waveform generator is considerable, it is worth noting that the microwave signal for the random-phase OFC can also be generated using a low-cost field-programmable gate array (FPGA)^34^. Incorporating an FPGA for generating the dual OFC may further enhance the advantage of the low-bandwidth detection stage provided by our work.

As a somewhat related concept, it is worth mentioning that there have been reports on BOTDA systems utilizing multiple spectral lines (or tones) for potential enhancement of sensing speed. In these cases, the line spacing between tones is set to be larger than the Brillouin gain bandwidth and a concatenation of pump pulses with different center frequencies is used in the interaction. The obtained system allows avoiding the required step-by-step frequency sweep between the pump and probe waves^35^.

In a more fundamental side, it should be stressed that this is the first time that the time expansion scheme is used advantageously to measure a nonlinear process like SBS. We believe that similar ideas could be exploited in other nonlinear measurement schemes involving the nonlinear beating of two optical waves. Introducing time expansion schemes in optical nonlinear measurements could yield revolutionary results, much in the same way as the use of dual-comb techniques were revolutionary in optical spectroscopy.

## Materials and methods

In this section, we present the theoretical analysis of a BETDA system. For this purpose, let us consider the propagation of two counterpropagating pump and probe beams with complex amplitudes *A*_P_ and *A*_S_, and frequencies *f*_0_ + Δ*ν* and *f*_0_, respectively. Δ*ν* is the frequency offset between the two waves which is assumed to lie in the vicinity of *ν*_B_. The electric fields *E*_P_ and *E*_S_ of pump and probe beams and the density variation *ρ*′ are expressed by:

1 $${E}_{{\rm{P}}}(z,t)={A}_{{\rm{P}}}(z,t)\cdot {e}^{i[2\pi ({f}_{0}+\varDelta \nu )t-{k}_{{\rm{P}}}z]}$$

2 $${E}_{{\rm{S}}}(z,t)={A}_{{\rm{S}}}(z,t)\cdot {e}^{i[2\pi {f}_{0}t+{k}_{{\rm{S}}}z]}$$

3 $$\rho {^{\prime}} (z,t)=Q(z,t)\cdot {e}^{i[\varOmega t-{k}_{A}z]}$$

where *A*_P_ and *A*_S_ are slowly varying amplitudes that may include external modulation terms. The signs used in the equations imply that the pump at *f*_0_ + Δ*ν* enters the fiber at *z* = 0 and propagates towards *z* = *L* while the probe is inserted at *z* = *L* and propagates towards *z* = 0.

The Brillouin interaction in these conditions can be described with a set of three coupled amplitude equations describing, respectively, the evolution of the two optical beams (*A*_P_, *A*_S_) and the acoustic wave *Q*^36^:

4 $$\frac{\partial {A}_{\rm{P}}}{\partial z}+\frac{{n}_{{\rm{g}}}}{c}\frac{\partial {A}_{\rm{P}}}{\partial t}=i\frac{{g}_{2}}{2}{A}_{\rm{S}}Q$$

5 $$\frac{\partial {A}_{\rm{S}}}{\partial z}-\frac{{n}_{\rm{g}}}{c}\frac{\partial {A}_{\rm{S}}}{\partial t}=-i\frac{{g}_{2}}{2}{A}_{\rm{P}}{Q}^{\ast }$$

6 $$\frac{\partial Q}{\partial t}+{V}_{\rm{A}}\frac{\partial Q}{\partial z}+{\varGamma }_{\rm{A}}(z,\varDelta \nu )Q=i{g}_{1}{A}_{\rm{P}}{{A}_{\rm{S}}}^{\ast }$$

where *g*_1_ and *g*_2_ are, respectively, the electrostrictive and elasto-optic coupling coefficients, *V*_A_ is the acoustic velocity, and *Γ*_A_ is a complex damping rate for acoustic wave that is a function of position and Δ*ν*, as described by the following formula:

7 $${\varGamma }_{\rm{A}}(z,\varDelta \nu )=\frac{{\varGamma }_{\rm{B}}}{2}+i\cdot 2\pi [{\nu }_{\rm{B}}(z)-\varDelta \nu ]$$

where *Γ*_B_ is an acoustic damping constant. Note that, for simplicity, the optical losses have been neglected and the Brillouin characteristics of the fiber have been assumed constant all along its length. The effects of cross-phase modulation (XPM) and self-phase modulation (SPM) are neglected by assuming that the peak powers are relatively low. In our derivation using perturbation theory, we will assume that the acousto-optic coupling terms in Eqs. (4) and (5) are comparatively weak, i.e., the Brillouin gain on the probe is small and the depletion of the pump is essentially negligible.

We start by considering the case of an ordinary BOTDA system for comparison, where the pump is pulse-modulated and the probe is a continuous wave. The pulse repetition rate *f*_R_ is selected to ensure *f*_R_ < *c*/2*n*_g_*L*, where *c*, *n*_g_, *L* being the speed of light in vacuum, the group refractive index, and the fiber length, respectively. The repeated pulse trains are analogous to an OFC where frequency components are spaced by *f*_R_ and share the same phase. The amplitude of pump and probe can be described by:

8 $${A}_{\rm{P}}=\mathop{\sum }\limits_{m=-N/2}^{+N/2}{A}_{\rm{P},m}\cdot {e}^{i2\pi m{f}_{\rm{R}}t}$$

9 $${A}_{\rm{S}}={A}_{\rm{S0}}$$

where *N* is the number of significant spectral lines within the pulse spectrum. Note that the spatial resolution is determined by the pulse duration, which is inversely related to the spectral width of the pump, given by *N·f*_R_. If Eqs. (6), (8) and (9) are applied to Eq. (5) assuming quasi-static acoustic wave by ignoring the derivative terms in Eq. (6), we arrive at a differential equation that describes the intensity change of the probe concerning both time and position:

10 $$\begin{array}{l}\frac{\partial {I}_{\rm{S}}}{\partial z}={A}_{{\rm{S}}}^{\ast }\frac{\partial {A}_{{\rm{S}}}}{\partial z}+{A}_{{\rm{S}}}\frac{\partial {A}_{{\rm{S}}}^{\ast }}{\partial z}\\ \qquad=-\mathop{\sum}\limits_{m}\mathop{\sum}\limits_{m{^{\prime}} }g(z,\varDelta \nu +(m-m{^{\prime}} ){f}_{{\rm{R}}})\cdot {A}_{{\rm{S}}0}{A}_{{\rm{S}}0}^{\ast }{A}_{{\rm{P}},m}{A}_{{\rm{P}},m{^{\prime}} }^{\ast }\cdot {e}^{i[2\pi {f}_{{\rm{R}}}t(m-m{^{\prime}} )]}+{{\rm{c}}}{{.\rm{c}}}{{.}}\end{array}$$

where *g* (*z*, Δ*ν* + (*m*–*m*′)·*f*_R_) = *g*_1_ *g*_2_ / Γ_A_(*z*, Δ*ν* + (*m*–*m*′)·*f*_R_) is a complex Brillouin gain according to position and detuning factor. Notably, due to the inclusion of the detuning factor (*m*–*m*′)·*f*_R_ in the Brillouin gain, each frequency component of the probe can exhibit a significant Brillouin gain, even when Δ*ν* is considerably different from *ν*_B_. This phenomenon underlies the broadening of the BGS acquired by a BOTDA system when the pulse spectrum significantly exceeds the intrinsic Brillouin gain bandwidth of approximately 30 MHz.

Subsequently, we examine the scenario of a BETDA system employing two OFCs in the pump and probe. In the spectral domain, *A*_P_ and *A*_S_ represent two intensity-modulated OFCs with line spacings of *f*_R_ + *δf* and *f*_R_, respectively. It is assumed that these combs share the same amplitude and spectral phase. In essence, both waves possess identical periodic modulation, differing only in a slight offset within their respective periods. The repetition rate *f*_R_ + *δf* is selected to ensure *f*_R_ + *δf* < *c*/2*n*_g_*L*. It will be assumed that *f*_R_ + *δf* is significantly smaller than the Brillouin gain bandwidth (~30 MHz) while the bandwidths of *A*_P_ and *A*_S_ largely exceed this value. As we will see later in the experimental part, typical values of *f*_R_ and *δf* are in the hundreds of kHz and Hz ranges while typical values of bandwidth of *A*_P_ and *A*_S_ are in the range of several GHz.

The pump and probe are expressed by:

11 $${A}_{{\rm{P}}}=\mathop{\sum }\limits_{m\,=\,-N/2}^{+N/2}{A}_{{\rm{P}},m}\cdot {e}^{i(2\pi m{f}_{{\rm{R}}}t+2\pi m\delta ft+{\phi }_{m})}$$

12 $${A}_{{\rm{S}}}=\mathop{\sum }\limits_{q\,=\,-N/2}^{+N/2}{A}_{{\rm{S}},q}\cdot {e}^{i(2\pi q{f}_{{\rm{R}}}t+{\phi }_{q})}$$

where *N* is the number of spectral lines of OFCs, and *ϕ*_*m*_ (*ϕ*_*q*_) is the phase for each frequency component of pump (probe) OFC. When Eqs. (6), (11), and (12) are put into Eq. (5) assuming quasi-static acoustic wave by ignoring the derivative terms in Eq. (6), the resulting differential equation for the probe intensity is as follows:

13 $$\begin{array}{c}\frac{\partial {I}_{{\rm{S}}}}{\partial z}=-\mathop{\sum}\limits_{q}\mathop{\sum}\limits_{q{^{\prime}} }\mathop{\sum}\limits_{m}\mathop{\sum}\limits_{m{^{\prime}} }g(z,\varDelta \nu +(m-m{^{\prime}} +q-q{^{\prime}} ){f}_{{\rm{R}}}+(m-m{^{\prime}} )\delta f)\\ \times {A}_{{\rm{S}},q}{A}_{{\rm{S}},q{^{\prime}} }^{\ast }{A}_{{\rm{P}},m}{A}_{{\rm{P}},m{^{\prime}} }^{\ast }\cdot {e}^{i[2\pi {f}_{{\rm{R}}}t(m-m{^{\prime}} +q-q{^{\prime}} )+{\phi }_{m}-{\phi }_{m{^{\prime}} }+{\phi }_{q}-{\phi}_{q{^{\prime}} }+2\pi \delta ft(m-m{^{\prime}} )]}+{{\rm{c}}}{{.}}\,{{\rm{c}}}{{.}}\end{array}$$

Equation (13) suggests that multiple frequency components contribute to the probe’s intensity variation through Brillouin gain. Simplification of this equation is possible by reducing the detection bandwidth (*f*_D_) to meet the condition of *N·δf* < *f*_D_ < *f*_R_*/*2. This process involves the elimination of frequency components beyond *f*_R_*/*2, and the remaining terms correspond to the selection of *m–m*′+*q*–*q*′ = 0. The differential equation then reduces to:

14 $$\begin{array}{c}\frac{\partial {I}_{{\rm{S}}}}{\partial z}=-\mathop{\sum}\limits_{q}\mathop{\sum}\limits_{q{^{\prime}} }\mathop{\sum}\limits_{m}\mathop{\sum}\limits_{m{^{\prime}} }g(z,\varDelta \nu +(m-m{^{\prime}} )\delta f)\\ \times {A}_{{\rm{S}},q}{A}_{{\rm{S}},q{^{\prime}} }^{\ast }{A}_{{\rm{P}},m}{A}_{{\rm{P}},m{^{\prime}} }^{\ast }\cdot {e}^{i[2\pi \delta ft(m-m{^{\prime}} )+{\phi }_{m}-{\phi }_{m{^{\prime}} }+{\phi }_{q}-{\phi }_{q{^{\prime}} }]}+{{\rm{c}}}{{.}}\,{{\rm{c}}}{{.}}\end{array}$$

Equation (14) notably exhibits the same time dependence as Eq. (10) derived for standard BOTDA systems, with the exception that *f*_R_ is substituted by *δf*. This replacement implies an expansion of the time scale by a factor of *f*_R_ */ δf* due to the introduction of the dual OFCs. Another noteworthy observation is the spectral width of the BGS, which is influenced by the spectral components in the probe comb lines experiencing non-negligible Brillouin gain from different orders of pump comb lines. The phase term *ϕ*_*m*_–*ϕ*_*m*_′+*ϕ*_*q*_–*ϕ*_*q*_′ plays a crucial role in determining the effective amount of Brillouin gain for a given probe component *q*. For instance, when a constant phase distribution (i.e. pulse modulation) is employed for the dual OFCs, all the spectral components share a common phase, resulting in *ϕ*_*m*_–*ϕ*_*m*_′+*ϕ*_*q*_–*ϕ*_*q*_′ = 0. This leads to a significant amount of Brillouin gain from different orders of the pump comb lines *m* and *m*′ satisfying *m–m*′+*q*–*q*′ = 0 in Eq. (14).

In contrast, the situation differs for a random phase distribution. It is important to note that the pump and probe spectra are stretched copies of the same comb spectra, with the same arbitrary phase for each comb line. For a given probe component *q*, the phase term *ϕ*_*m*_–*ϕ*_*m*_′+*ϕ*_*q*_–*ϕ*_*q*_′ is assumed to be random for various combinations of *q*′, *m*, and *m*′ that satisfy *m–m*′+*q*–*q*′ = 0. The random phases cancel out the Brillouin gain, except for two cases: (*m*′ = *q*, *m* = *q*′) and (*q*′ = *q*, *m* = *m*′), leading to *ϕ*_*m*_–*ϕ*_*m*_′+*ϕ*_*q*_–*ϕ*_*q*_′ = 0. The former corresponds to the beating between the pump and probe comb lines of the same order, and the latter is the DC component in Eq. (14). Consequently, one can obtain a narrowband local BGS through the use of a random phase distribution in the comb lines. Simultaneously, the spatial resolution is expected to align with that of BOTDA, which utilizes a pulsed pump possessing a power spectrum identical to the OFC.

**Fig. 7:**
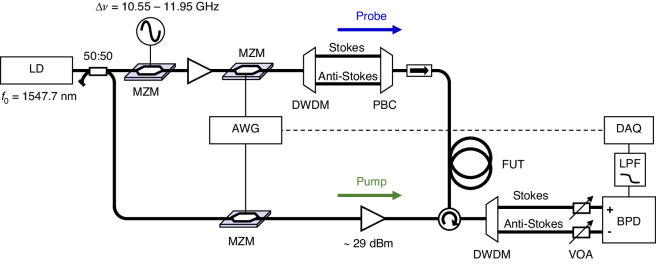
Setup of the Brillouin Expanded Time Domain Analysis (BETDA) system: LD laser diode, MZM Mach-Zehnder modulator, AWG arbitrary waveform generator, PBC polarization beam combiner, BPD balanced photo detector, VOA variable optical attenuator, LPF low pass RF filter, DWDM dense wavelength division multiplexing filter, DAQ data acquisition

Figure 7 shows the experimental setup of the BETDA system. A 1547.7 nm laser diode (LD) served as light source. Its output was split into probe and pump branches through a 50:50 coupler. In the probe arm, a microwave generator and a Mach-Zehnder modulator (MZM) were employed to generate two sidebands with a frequency offset of Δ*ν* in the vicinity of *ν*_B_. The bias point of the MZM is set to the maximum suppression of the optical carrier. The modulated output was amplified to 13 dBm and its intensity was modulated with a random-like waveform through another MZM. The modulating waveform is synthesized offline in the spectral domain, ensuring spectral symmetry around zero frequency and forcing all the comb spectral lines to have an arbitrary phase^26^. The resulting spectrum is transformed into a real-valued electrical signal by inverse Fourier transform, and loaded as time-domain samples in an arbitrary waveform generator (AWG). The optical comb spectrum was split into lower frequency (Stokes) and higher frequency (anti-Stokes) bands by a dense wavelength division multiplexing (DWDM) filter, and subsequently merged by a polarization beam combiner (PBC) and launched into a fiber under test (FUT) through an optical isolator. The lengths of the optical paths between the DWDM filter and the PBC were equalized to implement the scheme of orthogonally polarized probe sidebands (OPS)^37,38^. The anti-Stokes band is injected with a polarization orthogonal to the Stokes band. Such scheme compensates for the polarization dependence of Brillouin gain through the balanced detection.

In the other arm, the pump was also intensity-modulated by another MZM with the same intensity and phase profile as used in the probe arm, except for a small frequency offset (*δf*) in the line spacing of the OFC, resulting in a slightly shorter period of the output waveform. The pump comb was amplified to 29 dBm by a high-power optical amplifier and then launched into the FUT via an optical circulator. Following Brillouin interaction in the FUT, the OPS were separated by another DWDM filter and measured by a balanced photo detector (BPD) to obtain fully polarization-compensated traces. A low-pass RF filter (LPF) with a cutoff frequency of *f*_R_ /2 or less eliminated unwanted signals caused by beating between comb lines of staggered order within the detection band. Additionally, a square wave at *δf*, corresponding to the difference in the repetition rates of two OFCs, served as a trigger for measuring the time-expanded probe traces.
